# Increased JNK1 Signaling Pathway Is Responsible for ABCG2-Mediated Multidrug Resistance in Human Colon Cancer

**DOI:** 10.1371/journal.pone.0041763

**Published:** 2012-08-01

**Authors:** Ming Ming Zhu, Jin Lu Tong, Qi Xu, Fang Nie, Xi Tao Xu, Shu Dong Xiao, Zhi Hua Ran

**Affiliations:** Division of Gastroenterology and Hepatology, Shanghai Jiao-Tong University School of Medicine, Renji Hospital, Shanghai Institute of Digestive Disease; Key Laboratory of Gastroenterology and Hepatology, Ministry of Health (Shanghai Jiao-Tong University), Shanghai, China; University of Navarra, Spain

## Abstract

Multidrug resistance remains a major obstacle to effective chemotherapy of colon cancer. ABCG2, as a half-transporter of the G subfamily of ATP-binding cassette transporter genes (ABC transporters), is known to play a crucial role in multidrug resistance. However, the molecular mechanism of controlling ABCG2 expression in drug resistance of colon cancer is unclear and scarcely reported. In the present study, we systematically investigate the potential role of the c-Jun NH2-terminal kinase (JNK) signal pathway in ABCG2-induced multidrug resistance in colon cancer. In the hydroxycamptothecin (HCPT) resistant cell line SW1116/HCPT from human colon cancer cell line SW1116, ABCG2 is the major factor for multidrug resistance, other than well-studied ABCB1 or ABCC1. Our findings indicate that blocking the JNK pathway by pathway inhibitor SP600125 reduces the expression level and transport function of ABCG2 in drug-resistant cells SW116/HCPT. Notably, the experiments of small interfering RNA directed against JNK1 and JNK2 show that only silence of JNK1 gene has the equal effect as SP600125 on dephosphorylation of transcription factor c-Jun and the expression of ABCG2 protein, while the corresponding phenomena were not observed after silence of JNK2 gene. Meanwhile, SP600125 induces the apoptosis of SW116/HCPT cells by promoting the cleavage of PARP and suppressing the anti-apoptotic protein survivin and bcl-2, and increases the sensitivity of SW1116/HCPT to HCPT. Taken together, our work demonstrated that JNK1/c-jun signaling pathway was involved in ABCG2-mediated multidrug resistance in colon cancer cells. Definitely, inhibition of the JNK1/c-jun pathway is useful for reversing ABCG2-mediated drug resistance in HCPT-resistant colon cancer cells.

## Introduction

Multidrug resistance, by which cells resist many structurally and functionally unrelated drugs, is a major obstacle to effective chemotherapy of cancer. As for the mechanisms of multidrug resistance, the most important one is that accumulation of drug within cells is decreased by reduced inward transport or increased drug efflux, such as overexpression of adenosine triphosphate (ATP)-binding cassette (ABC) transporters [1]. Naturally, deeply understanding molecular mechanism of transporter expression has attracted intensely concentration for more successful therapeutic protocols underlying drug resistance.

In the human genome, 48 different ABC transporters have been identified and divided into seven subfamilies (A-G) based on the sequence similarities [2]. ABCG2, as a half transporter member of the ABCG subfamily, is a 655 amino acid protein that contains an ATP-binding domain and six transmembrane domains [3]. ABCG2 is originally identified in anticancer drug-resistant human cancer cell lines by in vitro selection [4]–[6]. As well as to the well-studied multidrug resistant protein P-glycoprotein(ABCB1), the overexpression of ABCG2 results in cancer cells resistant to various chemotherapeutic drugs by extruding these compounds out of the cells, such as topotecan and methotrexate [7], [8]. In our previous research, both the gene chip and the real-time PCR results based on the SW1116/HCPT cells indicated that the expression of ABCG2 increased significantly in contrast with the parental SW1116 cells [9]. Specifically, the mRNA expression of ABCG2 in the SW1116/HCPT cells enhanced more than 200 times in contrast with the parental SW1116 cells, while other transporters such as ABCB1, ABCC2, ABCC3 and ABCC6, increased only 0.5–1.0 times. The results implied that ABCG2 should play a major role in resistance of SW1116/HCPT cells to hydroxycamptothecin (HCPT), an inhibitor of topoisomerase I and less toxic than camptothecin. However, the molecular mechanism of ABCG2 expression and regulation in drug resistance is not clear and unanswered.

Aside from overexpression in multidrug-resistant cancer cells, ABCG2 is also widely expressed in a variety of normal tissues including in the epithelium of the small intestine, the liver canalicular membrane and ducts and lobules of the breast [10]. Moreover, ABCG2 plays an important role in the maintenance of the stem cell phenotype correlating with poor prognosis of chemotherapy [11]. Initial characterization of the ABCG2 promoter reveals that it is TATA-less with multiple activator protein 1 (AP1) binding sites [12].

C-Jun NH2-terminal kinase (JNK) is a member of the mitogen-activated protein kinase family that bind the NH2-terminal activation domain of the transcription factor c-Jun as a crucial member of AP1 family. The activity of JNK has been implicated in the regulation of cell proliferation, apoptosis and tumor transformation. Importantly, the previously reports revealed that modulation of JNK activation may be a novel method to reverse multidrug resistance, through regulation the expression level of multidrug resistance protein, such as P-glycoprotein(ABCB1) and MRP1(ABCC1) [13]–[16]. However, the relationship of JNK activation and ABCG2 expression is scarcely studied, particularly in colon cancer cells. Recently, it was reported that cancer cells with ABCG2-overexpressing had obvious change in endogenous JNK activity [17], [18]. And different topoisomerase inhibitors were found to activate JNK and to induce cell apoptosis [19]–[21]. With these minds, we focused on the expression linkage between ABCG2 and the involvement of JNK/c-Jun in the HCPT-resistant colon cancer cell subline SW1116/HCPT. Below, the results of our studies demonstrate that inhibition of the JNK pathway is able to down-regulate ABCG2 and the JNK pathway can be exploited for overcoming ABCG2-mediated multidrug resistance in colon cancer.

## Materials and Methods

### Materials

RPMI1640 culture medium and fetal bovine serum (FBS) were purchased from GIBCO Life Technology (Gibco, GrandIsland, NY). MTT [3-(4, 5-dimethyl thiazol-2-yl)-2, 5-diphenyl tetrazolium bromide], dimethyl sulfoxide (DMSO), BSA, fumitremorgin C (FTC), Hoechst33342, propidium iodide (PI) and SP600125 were purchased from Sigma (St. Louis, MO). HCPT was purchased from Tiancheng Pharmaceutical Co. (Changchun, China). Antibodies specific for rabbit JNK, P-JNK (Thr183/tyr185), c-Jun, P-c-Jun (Ser63), survivin, bcl-2, PARP were obtained from Cell Signaling Technology (Beverly, MA). Mouse monoclonal antibody against ABCG2 was purchased from Santa Cruz (CA, USA). Rabbit polyclonal antibody against GAPDH, anti-rabbit IgG, anti-mouse IgG were obtained from Kangcheng Biotechnology (Shanghai, China). SP600125 were dissolved in DMSO.

### Cell Culture

The human colorectal cancer cell line SW1116 (obtained from Academy of Military Medical Science, Shanghai, China) and its HCPT-resistant subline SW1116/HCPT (established and maintained in our laboratory) [9], [22], [23], were cultured in flasks with RPMI1640 supplemented with 10% heat-inactivated FBS, 2 mmol/L L-glutamine, 100 U/ml penicillin, and 100 U/ml streptomycin at 37°C in a humidified atmosphere containing 5% CO_2_. SW1116/HCPT cells were established by increasing the concentration gradient of HCPT in a stepwise manner and displayed 120.0-, 48.2- and 2.1-fold resistance to HCPT, adriamycin and oxymatrine compared with their corresponding parental cells, respectively. SW1116/HCPT cells were maintained in the culture medium already described and in the presence of 100 µg/L HCPT and incubated in drug-free medium for at least one week before use.

### Small Interference RNA (siRNA) for JNK1 and JNK2

Specific JNK1/2 siRNA (JNK1: GACCAUUUCAGAAUCAGACUU; JNK2: GAUGCUAACU UAUGUCAGGUU) was designed according to the cDNA sequence of JNK1/2 [24]. The negative control siRNA (UUCUCCGAACGUGUCACGUTT) encoded sequences of the control siRNA have no significant homologization with human and mouse cDNA. The siRNA was synthesized by GenePharma company (Shanghai, China). SW1116 and SW1116/HCPT cells were transfected with 100 nM siRNA for 48 hours using Lipofectamine™ 2000 Transfection Reagent (Invitrogen, Carlsbad, CA) according to the manufacturer’s instructions. After siRNA transfection with JNK1/2 siRNA, JNK1/2 protein expression levels were detected by Western blot.

### Semiquantitative Reverse Transcription-PCR of ABCG2

Cells were treated with either 20 µM SP600125 or 100 nM JNK1/2 siRNA, and total RNA was then extracted using the TRIzol reagent (Invitrogen, San Diego, CA) according to the manufacturer’s instructions. RNA purity and yield were assessed by spectrophotometric analysis. cDNA synthesis was carried out using RNA LA PCR kit (Takara, Tokyo, Japan). Real-time PCR was carried out using SYBR Green PCR Master Mix and an ABI-7300H Sequence Detection System (Applied Biosystems). All experiments were performed in triplicate and normalized to the internal reference gene glycer-aldehyde-3-phosphate dehydrogenase (GAPDH). Relative mRNA expression were calculated using the 2^(−Delta Delta CT)^ method as described previously [25], where CT (cycle count) is the threshold cycle value.

### Western Blot Analysis

Whole cell lysate extracts were prepared, and protein concentrations were measured by the BCA Protein Assay Reagent (Pierce Biotechnology, Rockford, IL). Protein extracts (30 µg/lane) were separated by 10% sodium dodecyl sulfate-polyacrylamide gel electrophoresis (SDS-PAGE). After electrophoresis, protein was transferred onto a polyvinylidene difluoride membrane (Millipore, Bedford, MA). The membranes were blocked for 1 hour in Tris Buffered Saline (TBS) containing 0.1% Tween 20 and 5% non-fat powdered milk and then incubated first with primary antibodies at 4? overnight and then horseradish peroxidase-conjugated secondary antibodies for 1 hour at room temperature, respectively. Specific proteins were visualized with the ECL Western blotting system according to the manufacturer’s instructions (Pierce Biotechnology). To ensure similar protein loading, the membrane was probed with a monoclonal antibody specific for GAPDH.

### Efflux Assay

The well-known substrate for ABCG2, Hoechst 33342 was selected to evaluate the efflux assay of ABCG2. SW1116 and SW1116/HCPT cells were incubated with Hoechst 33342, and the proportion of cells that exclude Hoechst 33342 was evaluated by MoFlo (DakoCytomation, Fort Collins, CO, USA) [26]. The specific inhibitor of ABCG2, fumitremorgin C (FTC), was used as the positive control [27]. Briefly, cells were incubated (10*6 cells/ml) in prewarmed RPMI1640 (supplemented with 10% FBS) containing 5 µg/ml Hoechst 33342 at 37°C for 90 min with intermittent shaking. The control cells were incubated in the presence of 10 µM of FTC for 30 min. After incubation, cells were then washed in ice-cold PBS and resuspended in cold PBS containing 1 µg/mL PI to label dead cells for flow cytometric analysis. Only events from viable cells were used for data analysis. The Hoechst 33342 dye was excited at 355 nm, and its emission fluorescence was detected using 450 nm and 675 nm filter systems. All data were analyzed using FlowJo software (Tree Star, Ashland, OR, USA).

### Cell Proliferation and Viability Assays

The effects on cell proliferation were evaluated using the MTT assay. Different concentrations of drugs were used, ranging from 0.1 mg/L to 0.8 mg/L of HCPT for SW1116 cells, and 0.1 mg/L to 62.5 mg/L of HCPT for SW1116/HCPT cells. Briefly, cells were seeded in 96-well microtiter plates at an initial density of 3×10^3^ cells/well. After incubation for 24 hours, cells were incubated with 20 µM SP600125, serial dilutions of HCPT and compound of SP600125 and HCPT for designated periods of time (24 or 48 hours), respectively. For siRNA transfection experiments, cells were transfected with 100 nM siRNA, including no siRNA, negative siRNA, and siRNA targeting JNK1 and JNK2. After transfection of 12 hours, various concentrations of HCPT were added. Assays were performed at 24 and 48 hours post transfection according to the manufacturer’s protocols. Twenty microliters of MTT solution (1 mg/ml) as added to each well, and after 4 hours, 150 µL DMSO was added to each well and incubated for 10 minutes. Absorbance was measured by a microculture plate reader at 570 nm. Data represent mean ± SD from three independent experiments.

### Apoptosis Assay

Apoptosis was determined by flow cytometry analysis using annexin-V FITC and PI double staining assay in accordance with the manufacturer’s instructions (Jingmei, Shenzhen, China) as described earlier. Briefly, after treatment, both floating and trypsinized adherent cells were harvested and resuspended at density of 1×10^6^ cells/ml in 200 µL binding buffer containing 5 µL of annexin-V fluorescein isothiocyanate and 5 µL of PI, then incubated for 10 minutes in the dark at room temperature. Analysis was immediately performed using flow cytometer.

### Statistical Analysis

Statistical analyses were performed using SPSS 17.0 software (SPSS, Chicago, IL). Continuous data were presented as the mean ± standard deviation (SD) for at least 3 repeated individual experiments for each group. Statistical differences were determined by using ANOVA and Student’s t-test for independent samples. A value of *P*<0.05 was considered statistically significant.

## Results

### Resistant Cell Lines Present Increased Expression of ABCG2 and Higher Activity of JNK/c-jun Pathway

Among many different mechanisms to interpret the development of multidrug resistance phenotype in cancer cells, the most extensively studied one is based on ABC transporters. The HCPT-resistant subline SW1116/HCPT that was selected from SW1116 cell line with increasing concentrations of HCPT displayed multidrug resistance phenotype by their wide cross-resistance and defects in intracellular drug accumulation.

**Figure 1:**
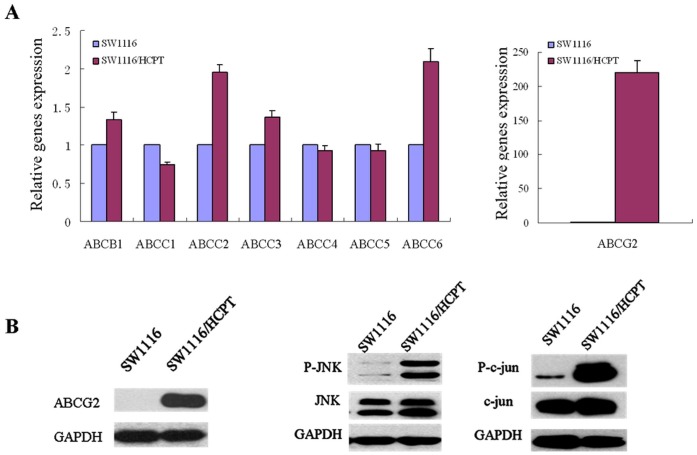
The expression of ABC transporters and activation of JNK/c-jun in parental and multidrug resistant cells. A. The mRNA expression of ABCB1, ABCC1–6, ABCG2 in SW1116 and SW1116/HCPT cells were measured by Real-time PCR. **B.** SW1116 and SW1116/HCPT cells were harvested to prepare cell lysates. The lysates were subjected to SDS–PAGE and blotted with antibodies. Levels of ABCG2, phosphorylated JNK (P-JNK), total JNK, phosphorylated c-jun (P-c-jun) and total c-jun were detected.

In Fig. 1A, SW1116/HCPT displayed obviously overexpression of ABCG2, whereas almost no ABCG2 expression was observed in the parental cell lines SW1116. While other transporters such as ABCB1, ABCC2, ABCC3 and ABCC6, increased only 0.5–1.0 times. It indicated that the multidrug resistant phenotype was mainly mediated by increased expression of ABCG2. Moreover, the level and activity of JNK pathway in SW1116/HCPT were examined after the cells were released to drug-free medium for 7 days. Obviously, the activity of the JNK and transcription factor c-jun increased markedly in the SW1116/HCPT than that in the parental lines (Fig. 1B).

### SP600125 and siRNA JNK1 Inhibit the Activation of JNK/c-jun Pathway

**Figure 2:**
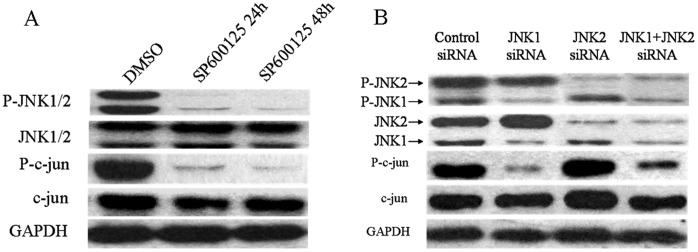
Effects of SP600125, JNK1 and JNK2 siRNA on the inhibition of the JNK/c-jun pathway in SW1116/HCPT cells. A. SW1116/HCPT cells exposed to DMSO and SP600125 at concentrations 20 µM for 24 and 48 hours, with medium replacement every 24 hours. Then expression of phospho-JNK/JNK and phospho-c-jun/c-jun was measured by Western blots. **B.** SW1116/HCPT cells were transfected with with control siRNA, siRNA JNK1 or siRNA JNK2 for 48 hours, then expression of phospho-JNK/JNK and phospho-c-jun/c-jun protein levels were measured by Western blot.

SP600125, as an inhibitor of the JNK pathway, reduced the P-JNK and P-c-jun expression markedly without modifying total JNK and c-jun expression (Fig. 2A). The small interfering RNAs (siRNAs) technology provides a powerful approach to targeted therapy of cancer. As previously reported, different JNK isoforms may have various functions. In our case, the small interfereing RNA targeting JNK1 and JNK2 that were ubiquitously produced were chosen to block the JNK1/2 signal pathway in SW1116/HCPT cells. Western blot analysis showed that the protein level of JNK1 and JNK2 decreased 72.3% and 78.4% after JNK1/2 siRNA transfection, compared with the control groups. And partial silence of JNK1 resulted in a significant decrease the activity of P-JNK1 and P-c-jun. However, partial silence of JNK2 only decreased phosphorylation of JNK2, but not phosphorylation of c-jun (Fig. 2B).

### Inhibition of JNK1/c-jun Pathway Down-regulates ABCG2 Expression Level

**Figure 3:**
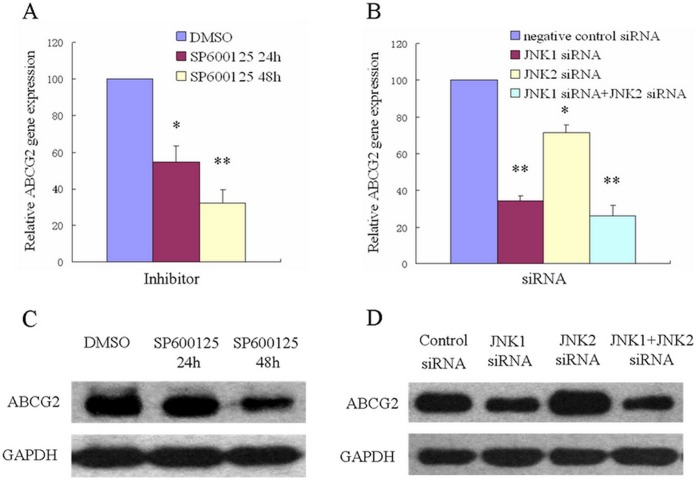
The expression of ABCG2 in the presence of SP600125 and silence of JNK1 or JNK2 genes in SW1116/HCPT. A and B. Real time-PCR analysis of ABCG2 gene expression in SW1116/HCPT cells by treatment without or with over a time course of SP600125 and JNK1**/**2 siRNA. *, *P*<0.05, **, *P*<0.01. **C and D.** The expression of ABCG2 protein was detected by Western-blot analysis after SP600125 and JNK1**/**2 siRNA treatment.

To examine whether ABCG2 was down-regulated by inhibition of JNK pathway at the mRNA level, ABCG2 mRNA expression by semiquantitative RT-PCR was analyzed. Upon treatment with 20 µM SP600125 for 24 or 48 hours in Fig. 3A, the ABCG2 mRNA expression in SW1116/HCPT cells was observed to be decreased about 45.42% and 67.83%, respectively. Furthermore, the suppressive effect of transfection of JNK1/2 siRNA was examined. Notably, silence of JNK1 exhibited a stronger effect on down-regulating ABCG2 mRNA expression than silence of JNK2 (Fig. 3B). The ABCG2 protein expression levels were then determined by Western blot analysis. As shown in Fig. 3C, SP600125 treatment for 48 hours suppressed ABCG2 protein expression 54.29%, indicating that inhibition of JNK pathway could down-regulate ABCG2 protein expression. Interestingly, compared with significant effect on down-regulating ABCG2 protein expression as silence of JNK1, there was no obvious effect for silence of JNK2 (Fig. 3D).

### Inhibition of JNK1/c-jun Pathway Inhibits the Efflux Assay of ABCG2

**Figure 4:**
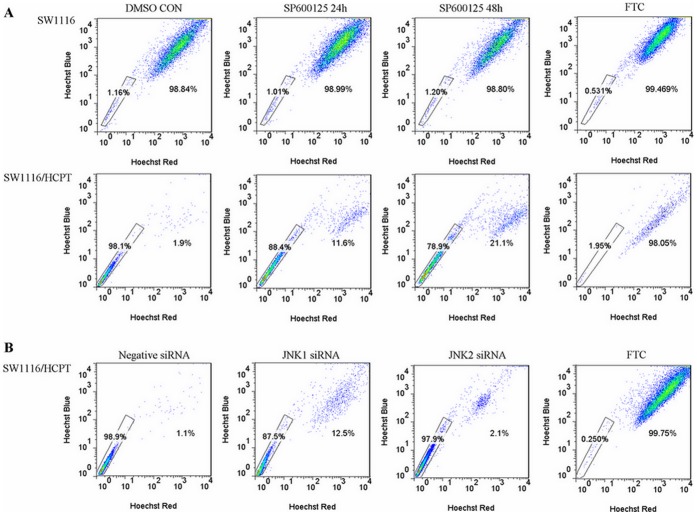
The function of JNK pathway inhibition on efflux assay of ABCG2. A. The intracellular fluorescence of Hoechst 33342 was analyzed by flow cytometer in SW1116 cells and SW1116/HCPT cells treated with or without SP600125. FTC as the positive control was incubated for 30 min. **B.** Hoechst 33342 was detected in SW1116/HCPT cells after tranfection with JNK1 or JNK2 siRNA for 48 hours. Box is Hoechst33342 staining-resistant area.

Our results pointed out SP600125 and JNK1 siRNA were able to down regulate the ABCG2 expression in SW1116/HCPT cells, so next we decided to evaluate the effect of inhibition of JNK pathway on ABCG2 efflux. For this purpose, Hoechst 33342 was determined as a measure of ABCG2 expression/function by flow cytometry. The known ABCG2 inhibitor, FTC, was used as a positive control. We found that SW1116/HCPT cells, which overexpressed ABCG2 gene, contained very low levels of Hoechst 33342 uptake in contrast with parent SW1116 cells. After treatment with SP600125 for 48 hours, Hoechst 33342 uptake is markedly increased in SW1116/HCPT cells. Whereas only 1.9% of SW1116/HCPT cells preferentially accumulated Hoechst 33342, this enhanced to 21.1% after treatment with SP600125 (Fig. 4A). Hoechst 33342 accumulation was also assessed in SW1116/HCPT cells after siRNA transfection with JNK1/2 siRNA for 48 hours. As shown in Fig. 4B, JNK1 siRNA transfection induced an increase in the proportion of cells that accumulate Hoechst 33342 (from 1.1% to 12.5%), while there was no obvious change of Hoechst 33342 accumulation by silence of JNK2 gene. In summary, flow cytometric analysis based on Hoechst 33342 indicated inhibition of JNK1 pathway could down-regulate the expression and transport activity of ABCG2.

### Inhibition of JNK1/c-jun Pathway Leads to Higher Apoptosis Induction in the Resistant Cell Lines

**Figure 5:**
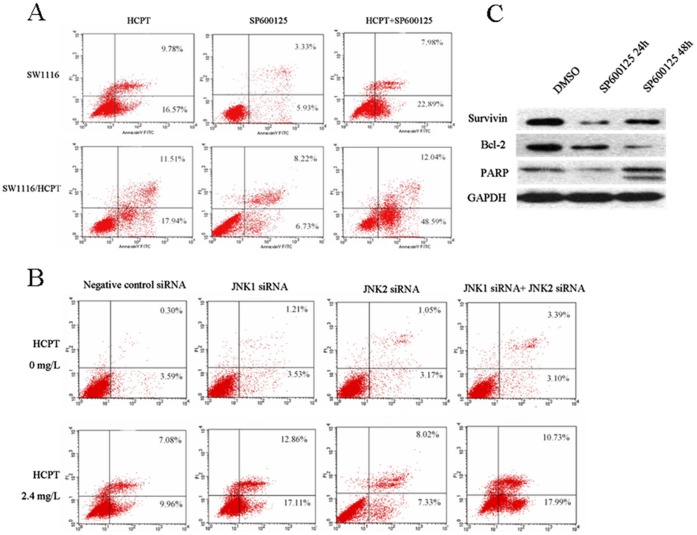
Apoptosis induction of JNK pathway inhibition in SW1116/HCPT cells. A. Apoptotic rates of SW1116 and SW1116/HCPT cells treated with indicated concentration of HCPT, with or without SP600125 for 48 hours, were analyzed by flow cytometry analysis after staining with Annexin V/PI. **B.** Apoptotic rates of SW1116/HCPT cells treated with combination of JNK1/2 siRNA and HCPT, were analyzed. **C.** The effect of SP600125 on the apoptosis regulator proteins in SW1116/HCPT cells were measured by Western-blot analysis.

To detect the role of the JNK/c-jun pathway in the survival of multidrug resistant cell lines, apoptosis induction after SP600125 treatment or JNK1/2 siRNA transfection was analyzed by flow cytometry analysis. The total apoptotic rate after SP600125 treatment 48 hours was 9.26% for parental SW1116 cells, while 14.95% for SW1116/HCPT cells (*P*<0.01). In addition, SP600125 and the silence of JNK1 gene could synergistically increase apoptosis induced by HCPT, instead of the silence of JNK2 gene. Compared with the control cells (0.1% DMSO and HCPT), there were significantly more apoptotic cells in SP600125 treatment group (SP600125 and HCPT) in SW1116/HCPT cells (60.63% vs. 29.45%), but not in SW1116 cells (30.87% vs. 29.45%) (Fig. 5A). In SW1116/HCPT cells, JNK1 siRNA transfection synergistically enhanced the apoptosis that induced by HCPT, comparing with the equivalent transfection with nonspecific siRNA (29.97% VS. 17.04%), while JNK2 has no remark effect on enhancement of the apoptotic rate (15.35% VS. 17.04%) (Fig. 5B). PARP cleavage as a key event of apoptosis and survivin, bcl-2 as markers for blocking apoptosis and regulateing cell division, were conducted by Western blot analysis. The result indicated that SP600125 could inhibit the expression of survivin and bcl-2 and induce the cleavage of PARP in resistant SW1116/HCPT cells (Fig. 5C). Thus, inhibition of JNK pathway induced the apoptosis of SW1116/HCPT cells by regulating the expression of apoptosis-related protein.

### Inhibition of JNK1/c-jun Pathway Increases the Sensitivity of SW1116/HCPT Cells to Anticancer Drugs

**Figure 6:**
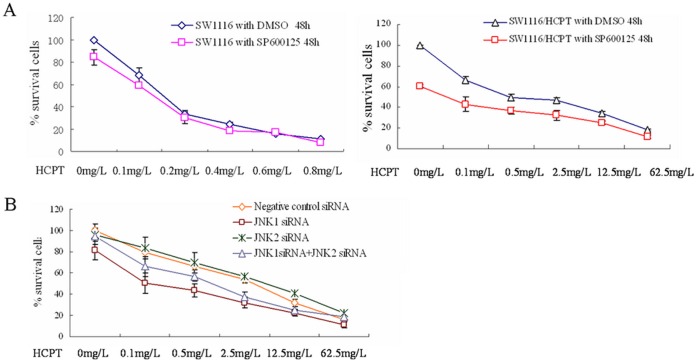
The functional role of JNK pathway inhibition on the sensitivity of SW1116/HCPT cells to HCPT. A. SW1116 and SW1116/HCPT cells were treated with indicated concentrations of HCPT, with or without SP600125, for 48 hours. Cell viability was determined by MTT assay. The percentage of viable cells was determined as described in the Materials and Methods section. **B.** Cell viability of SW1116/HCPT cells was determined by the MTT assay following treatment with JNK1, JNK2, or JNK1 and JNK2 siRNAs.

The effects of SP600125 and JNK1 siRNA on the ABCG2 protein level and on the accumulation of Hoechst 33342 suggested that JNK1 inaction might be able to sensitize the SW1116/HCPT cells to HCPT. We thus further assessed the efficacy of JNK signaling inhibitors in combination with HCPT. The MTT assay demonstrated that SP600125 can decrease the IC50 values of HCPT in the SW1116/HCPT cells. In Fig. 6A, after combination treatment with SP600125 for 48 hours, IC50 values of HCPT were decreased by >7-fold, from 2.41 mg/L to 0.328 mg/L in the SW1116/HCPT cells and from 0.154 mg/L to 0.142 mg/L in SW1116 cells. Similarly, JNK1 siRNA transfection also greatly increased the sensitivity of the SW1116/HCPT cells to HCPT by >7-fold, from 2.27 mg/L to 0.310 mg/L, while JNK2 siRNA transfection increased the resistance of SW1116/HCPT cells to HCPT (IC50 from 2.27 mg/L to 4.271 mg/L) on the contrary (Fig. 6B).

## Discussion

The effective chemotherapy is severely limited by multidrug resistance for patients who suffer from malignant tumors, including metastatic colorectal cancers. ABCG2 is an integral membrane protein as a drug efflux pump and is responsible for the “side population” phenotype of stem cells and seems to play a significant role in protection against hypoxia [28], [29]. Overexpression of ABCG2 can result in the acquisition of multidrug resistance to multiple anticancer drugs [7], [8]. Actually, our result demonstrated that constitutive overexpression of ABCG2 in drug-resistant colon cancer cells SW1116/HCPT resulted in great resistance to chemotherapeutic drugs, although the underlying molecular mechanisms remained unknown in detail. Thus, the strategy to reverse the multidrug resistant phenotype based on inhibition of ABCG2 expression or its transporter function by enhancing intracellular accumulation of anticancer drugs deserves to extensive and deeply investigation.

JNK is activated by proinflammatory cytokines, environmental stress (e.g., hypoxia, UV and g-radiation, heat shock, redox stress, etc.) or several cytotoxic drugs. Its activation generally contributes to the mediation of significant cellular events. The activity of JNK has been implicated in the regulation of embryonic morphogenesis, cell proliferation, tumor transformation, and apoptosis [30]–[33]. Most previously reports have identified the pro- or anti-apoptosis functions of JNK, depending on cell type, dosage of stimulus or duration of treatment and the activities of other cellular signaling pathways [34]–[38]. However, there is so far no consensus about the relationships between JNK pathway and expressions of transporters. Almost all previously studies were focus on P-glycoprotein-mediated multidrug resistance [15], [39], [40], but the relationship of JNK signal pathway and ABCG2 transporters is not reported in colon cancer cells.

In this work, the impact of JNK pathway on ABCG2 expression/activity and the implications on multidrug resistance mediated by these transporters were deeply and systematically investigated. Three JNK genes have been identified: JNK1, JNK2 and JNK3. JNK1 and JNK2 are ubiquitously expressed, whereas JNK3 is mainly expressed in the brain, testis and heart [41]. So the roles of either JNK1 or JNK2 in the program of multidrug resistance were all investigated in this study.

SP600125 is a selective inhibitor of the JNK/c-jun signaling pathway, which can display 20-fold higher specificity for JNK1 kinase relative to other kinases [42]. Importantly, the role of SP600125 on reversing the ABCG2-mediated multidrug resistance of SW1116/HCPT cells was observed in our experiment. As shown in Fig. 1B, phosphorylation of JNKs was increased in SW1116/HCPT cells. Meanwhile, c-jun activity was significant attenuated by SP600125 or after transfection with the JNK1 siRNA in Fig. 2. We also found that inhibition of JNK by SP600125 markedly decreased mRNA level, protein level and transport activity of ABCG2 in SW1116/HCPT cells.

To discriminate the effect of JNK1/2 signaling pathway on ABCG2 transporter, more comparative experiments were carried out. As expected, the blockade of JNK1 activity using JNK1 siRNA down regulated the expression of transport protein ABCG2 and the viability of SW1116/HCPT cells as did SP600125, but not blockade of JNK2 activity. Though the mRNA expression of ABCG2 was decreased after partial silence of JNK2, the protein expression was even elevated. The current data indicated that only JNK1 pathway promoted resistance to HCPT and positively regulates ABCG2 protein expression in multidrug resistant cells, but not JNK2 pathway. As previous reported, activation of JNK pathway was positively correlated with activation of the transcription factor, c-Jun. In our case, we also found that phosphorylation of JNK1 and the activity of transcription factor c-jun were key factors to overexpression of the ABCG2 gene in drug-resistant cells SW1116/HCPT. We believed that c-jun, as one member of AP-1, is likely to play the role by affecting on the AP1 binding sites of ABCG2 promoter.

PARP is a DNA repair enzyme activated by DNA damage and the cleavage of PARP has been widely used as a biochemical marker of apoptosis [43]. Thus, we further studied PARP levels in SW1116/HCPT cells after exposure to SP600125 and silence of JNK1 or JNK2 gene. Western blot analysis revealed that the intensity of 89 kDa cleavage of PARP band was increased in the cells treated with SP600125, meanwhile the bcl-2 and survivin, as the anti-apoptosis protein, were decreased after the equal treatment. These results suggested that inhibition of JNK1 pathway efficiently caused cell death through the induction of apoptosis in multidrug resistance cells.

Recently, more evidences indicate that anticancer drugs activate many signal pathways, some of which are connected to the development of drug resistance of tumor cells [40], [44]. As above mentioned in our case, incubation with HCPT for 24 hours can also induce JNK phosphorylation in SW1116 cells, this result is supported by previous report that JNK was preferentially activated by HCPT in other cell lines [21]. All of these results suggest that the JNK1/c-jun signal pathway is involved in expression of the ABCG2 gene in colon cancer cells, and may contribute to chemoresistance.

In conclusion, we demonstrated that JNK1/c-jun pathway is involved in the multidrug resistance of colon cancer cells. As SW1116/HCPT cells presented higher JNK/c-jun activity than SW1116 cells, inhibition of this pathway resulted in less ABCG2 expression and higher apoptosis induction in the resistant cells. In addition, JNK1/c-jun inhibition correlates with the down-regulation of survivin and bcl-2 and PARP cleavage. Further investigation with other tumor models as well as *in vivo* studies helps to understand the role of JNK1/c-jun pathway in multidrug resistance. Although the different roles of JNK isoforms remain unclear in details until now, there is no doubt that JNK1/c-jun signaling cascade should be considered as an attractive target for therapeutic intervention in our research.
